# Intrathecal decompression versus epidural decompression in the treatment of severe spinal cord injury in rat model: a randomized, controlled preclinical research

**DOI:** 10.1186/s13018-016-0369-y

**Published:** 2016-03-22

**Authors:** Jian Zhang, Huili Wang, Chenggang Zhang, Weiguang Li

**Affiliations:** No.1 Department of Orthopedic Surgery, Tianjin Baodi Hospital, No.8, Guangchuan Road, Baodi District, Tianjin, 301800 China; Institute of Radiation and Radiation Medicine, Academy of Military Medical Sciences, No.27, Taiping Road, Haidian District, Beijing, 100850 China

**Keywords:** Spinal cord injury, Basso-Beattie-Bresnahan score, Decompression, Animal model, Pathophysiology

## Abstract

**Background:**

In the setting of severe spinal cord injury (SCI), there is no markedly efficacious clinical therapeutic regimen to improve neurological function. After epidural decompression, as is shown in animal models, the swollen cord against non-elastic dura and elevation of intrathecal pressure may be the main causes of aggravated neurologic function. We performed an intrathecal decompression by longitudinal durotomy to evaluate the neuroprotective effect after severe SCI by comparing with epidural decompression.

**Methods:**

Eighty-four adult male Sprague-Dawley rats were assigned to three groups: sham group (group S), epidural decompression (group C), and intrathecal decompression group (group D). A weight-drop model was performed at T9. The Basso-Beattie-Bresnahan (BBB) score was used to evaluate neurological function. Animals were sacrificed at corresponding time points, and we performed pathohistological examinations including HE staining and immunohistochemical staining (IHC) of glial fibrillary acidic protein (GFAP), neurocan, and ED1 at the epicenter of injured cords. Finally, the lesions were quantitatively analyzed by SPSS 22.0.

**Results:**

The mortality rates were, respectively, 5.55 % (2/36) and 13.9 % (5/36) in groups C and D, and there was no significant difference between groups C and D (*P* = 0.214). Compared with epidural decompression, intrathecal decompression could obviously improve BBB scores after SCI. HE staining indicated that more white matter was spared, and fewer vacuoles and less axon degradation were observed. The expression peak of GFAP, neurocan, and ED1 occurred at an earlier time and was down-regulated in group D compared to group C.

**Conclusions:**

Our findings based on rat SCI model suggest that intrathecal decompression by longitudinal durotomy can prompt recovery of neurological function, and this neuroprotective mechanism may be related to the down-regulation of GFAP, neurocan, and ED1.

## Background

Traumatic spinal cord injury (SCI) is a disastrous event for patients and families; the global incidence rate is estimated at 23 cases per million individuals [1]. Although prominent improvements have been made in acute medicine including surgical management and rehabilitation, which have significantly elevated survival rate and decreased long-term complications for individuals with SCI, significant neurological recovery has not been clinically obtained.

In the setting of severe SCI, multiple preclinical studies aimed at improving neurological recovery by attenuating the process of secondary injury have been conducted. These studies have also shown that pharmacological therapy and early surgical decompression could improve the neurological outcome. Unfortunately, clinical studies have not currently demonstrated markedly efficacious therapeutic regimens that improve neurological function for paraplegic or tetraplegic individuals using techniques that include early surgical decompression, local hypothermia, drugs, and electrical stimuli [2–4]. The improved degree of neurological function in complete SCI patients is relatively low; the proportion that improve from the American Spinal Injury Association (ASIA) A to ASIA B or C is less than 10 %, and there are nearly no patients that undergo neurological recovery from ASIA A to D or E [3, 4].

Although decompressive surgery is a very important strategy for maintaining adequate blood flow and perfusion to prompt neurologic recovery, the ensuing edema and hemorrhage may lead to expansion of the injured cord and increase spinal cord interstitial pressure (CIP) against the relatively non-elastic dura mater [5]. Jones et al. found cerebrospinal fluid (CSF) pressure differential cranial-caudal to injured site increased after SCI [6], and they also found swollen cord immediately occluded subarachnoid space in severe SCI [7].

These observations suggested that decompression only by removal of osseous and soft-tissue elements might not be adequate and should be further improved to restore normal CIP. Waleed et al. found a durotomy could lead to a dramatic CIP drop in a distraction SCI model in vitro [8].

To date, only one preclinical study has evaluated the effect of durotomy in mild SCI in vivo [9]. Here, we hypothesized that severe thoracic SCI in rats would imitate paraplegia, and a durotomy might show some efficacy in relieving the injury. To elucidate the neuroprotective mechanism of durotomy, parameters including Basso-Beattie-Bresnahan (BBB) scores, HE staining, and the expression of glial fibrillary acidic protein (GFAP), neurocan, and ED1 were analyzed.

## Methods

### Animals and allocation

Eighty-four adult, male Sprague-Dawley rats (200–250 g, 8 weeks) were used in this study and provided by the Experimental Animal Center of Academy of Military Medical Sciences (Beijing, China). All animals were maintained for 5–7 days before surgery in a temperature-regulated room (22–25 °C) on a 12-h light/dark cycle with free access to food and water. After surgery, each rat was housed individually.

The rats were randomly assigned to three groups: sham group (group S, *N* = 12), only laminectomy; epidural decompression group (group C, *N* = 36), only laminectomy and SCI, which is deemed as epidural decompression; and intrathecal decompression group (group D, *N* = 36), longitudinal durotomy after SCI. The rats were evaluated before surgery and at 4 h and 1, 3, 7, 14, and 28 days post injury (DPI).

All experiments were conducted with approval of the Institutional Animal Care and Use Committee of Beijing Institute of Radiation Medicine and adhered strictly to the NIH Guide for the Care and Use of Laboratory Animals. All surgery was performed under chloral hydrate anesthesia, and all efforts were made to minimize suffering. We certify that all applicable institutional and governmental regulations concerning the ethical use of animals were followed during the course of this research.

### Surgical procedure for animal model

All rats were anesthetized with chloral hydrate (300 mg/kg, i.p.). After successful anesthesia, the rats were situated in prone position on the operating table. A 5-cm posterior midline incision was made, and the paravertebral musculature was separated from the lamina of T7–11. The spinous processes of T8–10 were removed, and then a laminectomy was performed at T8–10. After the cord was exposed, the spinal column was rigidly immobilized. A calibrated glass guide tube was positioned perpendicular above the cord center. The cord was subjected to a collision of a 10 g stainless steel rod from 50 mm height, described by Allen [10]. Rats in group D immediately received a longitudinal durotomy 10 mm in length (approximately two levels) cephalic-caudal to the injured cord by microsurgical apparatus under a microscope; gelfoam was overlaid onto dural incision. The muscle and skin were sutured in layers. All rats were injected daily for three consecutive days with physiological saline (2 ml/100 g, i.p.) to prevent hypovolemia. Their bladders were manually pressed twice daily for urination until they re-established bladder reflexes.

### Locomotor function assessment

The BBB scores were used to evaluate movement, body support, and coordination ability. Three days before surgery, all the animals were trained, and animals with intrinsic motor dysfunction were excluded. All results were recorded by a video camera for subsequent blinded examination by two scorers.

### Tissue preparation

The rats were sacrificed at corresponding times (*N* = 5 in groups C and D) with an overdose of chloral hydrate (500 mg/kg, i.p.) and transcardially perfused with saline solution (0.9 %) followed by 4 % cold paraformaldehyde. The injured cords were carefully removed en bloc, then fixed in 4 % paraformaldehyde at 4 °C for 24 h, and then embedded in paraffin after dehydration.

Four consecutive transverse sections (5-μm thickness) were prepared in all rats with a Leica microtome (Leica RM2035, Germany) at epicenter. One section was stained with HE, and the other sections were used for immunohistochemical staining (IHC) analysis.

### IHC analysis

The expression levels of proteins were detected by IHC using monoclonal mouse anti-rat GFAP (1:400; Sigma, St. Louis, MO), monoclonal mouse anti-rat neurocan (1:200; Sigma, St. Louis, MO), monoclonal mouse anti-rat ED1 (1:200; Abcam, New Territories, Hong Kong), as described by Cattoretti et al. [11]. After deparaffinization, hydrogen peroxide incubation, microwave antigen retrieval, and incubation in 2 % goat serum (SP9001 kit, Zhongshan Golden Bridge, Beijing, China), the sections were separately incubated in corresponding primary antibodies overnight at 4 °C. On the following day, the sections were incubated with a biotinylated secondary antibody at room temperature for 1 h and then incubated in avidin-biotin peroxidase complex (SP9001 kit, Zhongshan Golden Bridge, Beijing, China) for 30 min. The sections were visualized by 0.025 % diaminobenzidine (SP9001 kit, Zhongshan Golden Bridge, Beijing, China). For negative controls, PBS was used instead of primary antibodies.

All sections were examined under a light microscope (Olympus BH-2, Tokyo, Japan) and photographed (Sony, CCD-IRISI, Tokyo, Japan) by a pathologist who was blinded to experimental conditions. IHC-positive cell counts were performed in four obviously expressed, highly magnified fields, and averages were determined in different groups.

### Statistical analysis

The statistical analyses were performed by SPSS 22.0. All data were reported as means ± standard deviation. Values of *P* less than 0.05 were considered statistically significant.

## Results

### Observations from the operations

An enormous contraction of the hind limbs and tail was observed after collision, and an intrathecal hematoma occurred immediately; the spinal cord swelled and obstructed the intradural cavity. After durotomy was performed, the contusive, hemorrhagic spinal cord with a dark purple appearance herniated more than half of the intradural cavity.

### Differences in mortality rate

The mortality rates were 8.33 % (1/12), 5.55 % (2/36), and 13.9 % (5/36) in groups S, C, and D, respectively, and there was no significant difference between groups C and D (Table 1). For mortality reason, one died due to unrecovery after anesthesia, five died from pulmonary bleeding, and two died from bladder bleeding by post-mortem analysis.

**Table 1:** Comparison of mortality rate between groups C and D

Groups	Survival	Mortality	Total	Mortality rate (%)
C group	34	2	36	5.55
D group	31	5	36	13.9
Total	65	7	72	9.7

By chi-square test, there was no significant difference between groups C and D for mortality rate (*P* = 0.214)

### Changes of neurological function

**Fig. 1:**
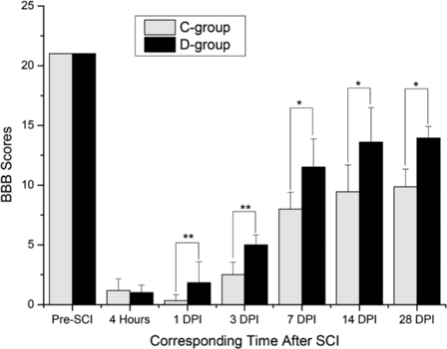
BBB score comparison between groups C And D. BBB scores were determined at corresponding time points before and after surgery in groups C and D; they were significantly different at 1, 3, 7, 14, and 28 DPI (*P < 0.05; **P < 0.001)

The BBB scores in each group all consisted of a value of 21 before the surgery (BBB scores in group S gradually recovered to normal levels at 1 DPI, not shown). The scores were sharply increased within 14 DPI in group D, but in group C, the scores were lower at 1 DPI, indicating neurological function was aggravated (Fig. 1).

### Histological results

**Fig. 2:**
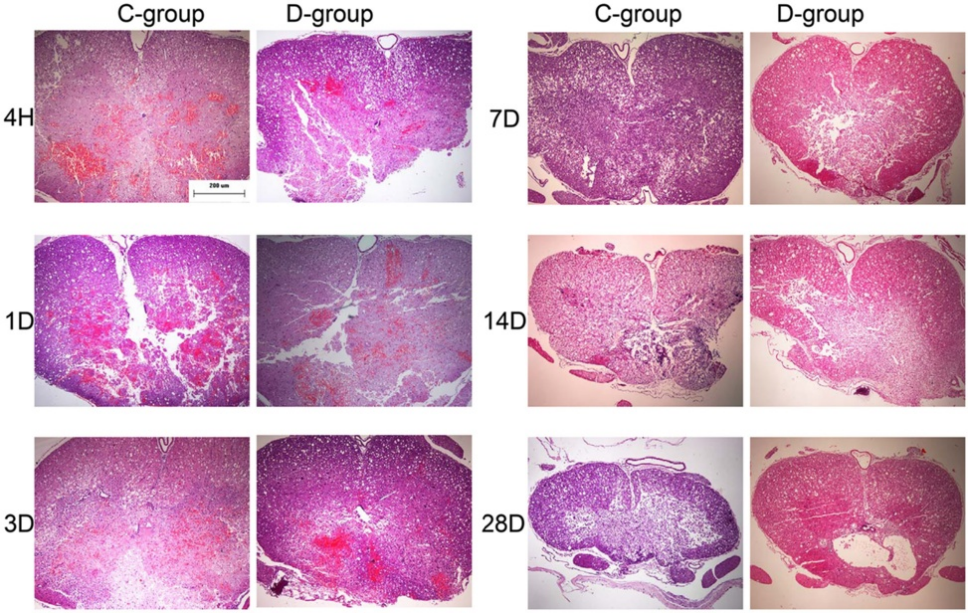
HE staining comparison between groups C and D. The pathological changes were similar between groups C and D from 4 h to 3 DPI. However, from 7 to 28 DPI, more white matter was spared and fewer vacuoles were observed in group D (HE staining, ×40; *bar* = 200 μm)

To analyze histological changes after SCI, HE staining was applied. No lesion was detected in group S, while irregular hemorrhage, neuron loss, vacuoles, axon degradation, and cavitation were observed in groups C and D (Fig. 2). More white matter was spared, and fewer vacuoles and less axon degradation were observed in group D (Fig. 2).

### IHC results

We performed IHC to elucidate the expression of GFAP, ED1, and neurocan. In group S, GFAP+ astrocytes without hypertrophy were observed, and there was no expression of neurocan and ED1. No staining was observed in negative control.

**Fig. 3:**
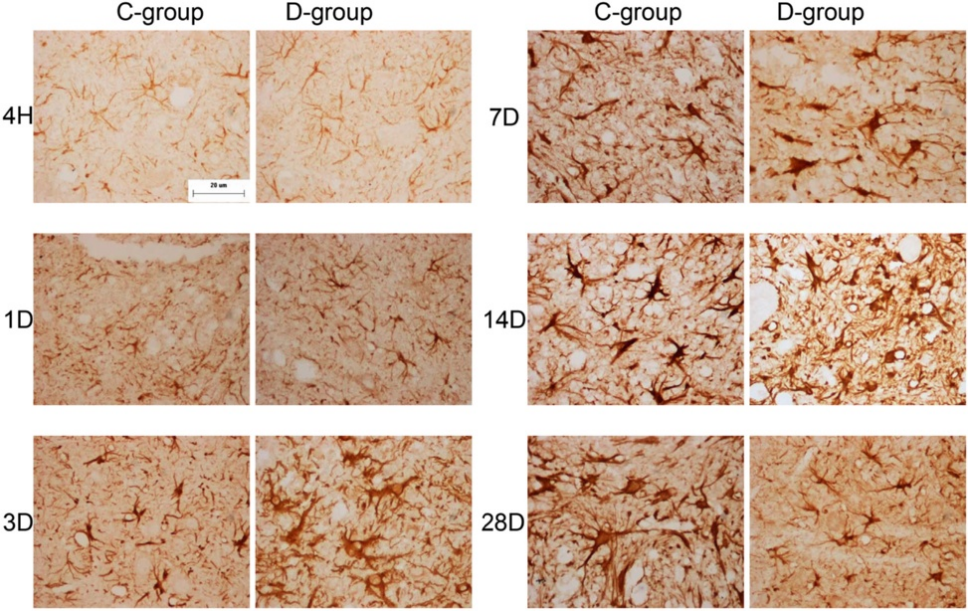
GFAP staining comparison between groups C and D. The peak of GFAP expression in group D occurred earlier than in group C, and GFAP expression was down-regulated compared to group C (*bar* = 20 μm)

The number of astrocytes did not increase in groups C and D compared to group S (Fig. 3), but the volume of astrocytes was significantly increased. GFAP+ astrocytes were detected mainly in gray matter. GFAP expression in group D increased sharply, reached peak values at 3 and 7 DPI, and then gradually declined from 14 to 28 DPI. However, GFAP expression in group C continuously increased from 3 to 28 DPI and reached peak values at 14 and 28 DPI.

**Fig. 4:**
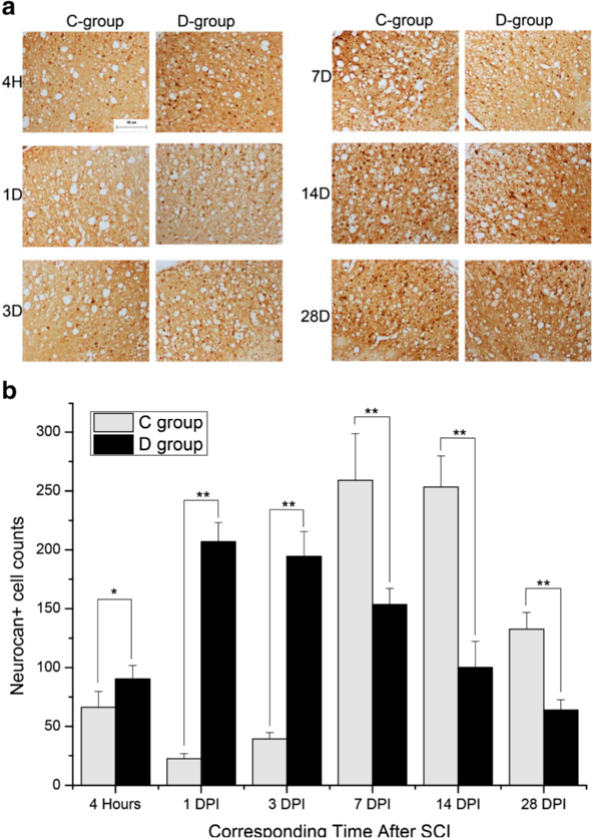
Neurocan staining comparison between groups C And D. Neurocan expression pattern was different in groups C and D, as shown in (**a**) (×200; *bar* = 40 μm). Positive cell counts were performed in highly magnified fields, as shown in **b** (*N* = 4; *P < 0.05; **P < 0.001)

Neurocan was mainly located in the white matter (Fig. 4). A two-peak expression pattern of neurocan occurred separately at 4 h and 7 DPI in group C, while in group D, neurocan expression reached a peak value at 1 DPI and then continuously decreased.

**Fig. 5:**
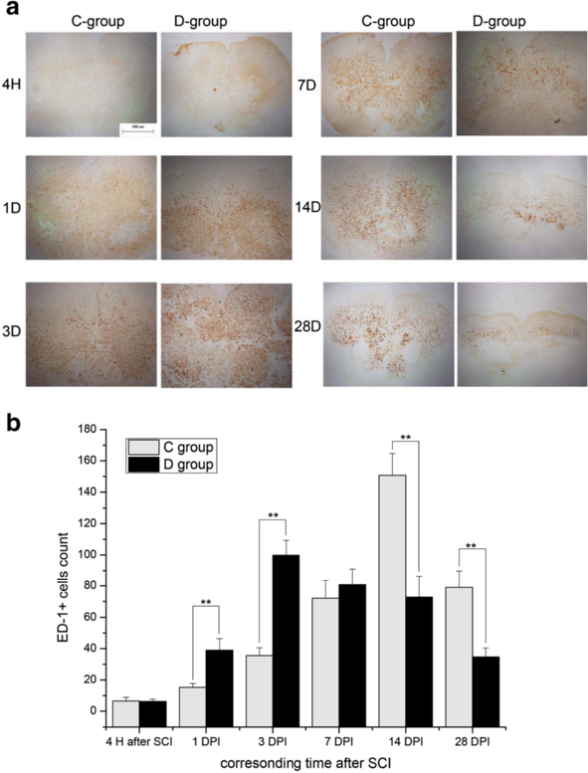
ED1 staining comparison between groups C And D. ED1-positive cells in group D was much higher than that in group C at 1 and 3 DPI, and equivalent at 7 DPI. However, it was much lower than that in group C at 14 and 28 DPI, as shown in (**a**) (×40; *bar* = 200 μm). The positive cell counts in highly magnified fields (×400) were significantly different at 1, 3, 14, and 28 DPI, as shown in (**b**) (*N* = 4; *P < 0.05; **P < 0.001)

ED1 was extensively distributed in the gray and white matter (Fig. 5). The expression peak of ED1 occurred at an earlier time and was much lower in group D than that in group C.

## Discussion

Epidural decompressive surgery is recognized as an important intervention and a general trend for treating SCI. Epidural decompression seems adequate during operation; however, postoperative magnetic resonance imaging often reveals that the swollen cord has filled subarachnoid space [12, 13]. Jones et al. found CSF pressure was only partially decreased by epidural decompressive surgery [6], and the enlarged cord immediately occluded subarachnoid space in severe SCI [7]. Smith et al. found the swollen cord partially herniated through incision site following durotomy [9]. These observations indicate that epidural decompression may not be adequate, and it is often necessary to perform further decompression after severe SCI.

Secondary injuries including edema and hemorrhage lead to an expanded volume of the injured cord against the non-elastic dura mater [5], leading to a circumstance similar to compartment syndrome, and ultimately worsening ischemia in the injured cord. Elevation of CSF pressure may worsen ischemia at the injured cord [14], and reduction of CSF pressure can enhance blood flow and improve microcirculation [15]. Obvious elevation of CSF pressure has been shown after SCI in preclinical and clinical studies [6, 15].

Only a few studies have examined durotomy use for the treatment of SCI. The possible mechanisms of the neuroprotective effect of a durotomy are as follows: a decrease in CIP [8], cavitation, scar formation, and lesional volume [9]; relief of congestive epidural veins; and restoration of CSF flow [16].

Due to the differences in injury types, severity, and levels, clinical manifestation of SCI is diverse, and none of the animal SCI models available can completely simulate the clinical situation, which may include fractures and dislocation. Furthermore, due to transportation, physical and radiographic examination, neurological function assessment, and the determination to perform surgery, patients cannot be surgically treated immediately after SCI [17]. This study was an ideal situation in which a durotomy was performed immediately to observe the neuroprotective effect.

Our SCI model imitated severe SCI, in which the obviously swollen injured cords against the dura were identified after collision. The neurological function was significantly improved after intrathecal decompression by longitudinal durotomy, and neurological recovery was in agreement with the results of pathohistological analysis in which more white matter was spared and fewer vacuoles and less axon degradation were observed. Our findings based on rat SCI model suggest that intrathecal decompression may be useful as a promising therapeutic regimen for SCI.

Damage to the spinal cord can result in a glial reaction and eventually glial scar formation, in which GFAP+ astrocytes and neurocan are important components in inhibiting neurological repair. In early stage, reactive astrocytes may exert beneficial effects by regulating local immune responses and promoting tissue repair; however, these beneficial effects arise at the expense of inhibiting damaged axon regeneration [18-20]. Neurocan is an inhibitor of central nervous system regeneration. Inhibition of neurocan expression can improve neurological repair [21]. In this study, GFAP+ astrocytes were hypertrophic with richly branched processes at the epicenter. However, after durotomy, GFAP expression peak occurred at an earlier time after SCI and was obviously down-regulated when comparing with epidural decompression. Neurocan was also down-regulated after durotomy. These results suggest that the neuroprotective mechanism of durotomy might be related to inhibit glial scar formation by down-regulating the expression of GFAP and neurocan at the injured site.

Because activated macrophages can release cytotoxic substances that aggravate inflammatory reaction in secondary injury [2], ED1 is a specific marker for macrophages. A key intervention is to control inflammatory reactions following SCI. We found that ED1 expression was down-regulated in group D compared to group C. Thereby, the durotomy might inhibit inflammatory reactions by suppressing the expression of ED1.

We also found that in the early stage after SCI, the expression of GFAP, neurocan, and ED1 were supppressed in group C. It is likely that the injured cord was functionally depressed and did not express corresponding proteins due to the compression from intact dura which possibly causes ischemia. However, we did not have the direct evidence which can show some degree of restoration of blood flow to the injured site after durotomy. Further studies need to be conducted to confirm whether blood flow can be restored by durotomy [22].

Maintenance of the dural integrity can inhibit inflammatory reaction and reduce scar formation [9, 23, 25]. However, there is no consensus on how to cover the neural tissues after durotomy. In studies by Smith [9] and Iannotti [23], they used a fibrin sealant to fix an allograft onto the incised dura. In a large animal model, Neulen et al. found that collagen matrix was an attractive alternative in duraplasty due to its easy handling, lower surgical time, and high biocompatibility [24]. However, when performing a decompressive craniectomy, the neurosurgeon usually applies nothing to cover cerebral tissue [25]. In this study, we applied gelfoam to cover the injured cord and did not find exacerbation of the inflammatory reaction or adhesive scar after a durotomy.

Although these results are encouraging, there are some limitations in this study. The mortality rate was increased after intrathecal decompression, which indicated that systematic pathophysiological reactions after durotomy were aggravated. We did not perform CIP measurements due to the small animal model. Further research upon durotomy need to be conducted to acquire more comprehensive data, for example, performing durotomy in large animal SCI model can better imitate the situation in human and evaluate the safety and efficacy of this procedure, investigation on microcirculation at the injured site can better understand the pathophysiological process of SCI, and suitable dural substitute can inhibit inflammatory reaction and reduce adhesive scar.

## Conclusions

Our findings based on rat SCI model suggest that intrathecal decompression can prompt recovery of neurological function which was in accordance with the pathohistological process, and this neuroprotective mechanism may be related to the down-regulation of GFAP, neurocan, and ED1. Therefore, intrathecal decompression may be useful as a promising therapeutic regimen for SCI.

## Abbreviations

ASIA: American Spinal Injury Association
BBB: Basso-Beattie-Bresnahan
CIP: spinal cord interstitial pressure
CSF: cerebrospinal fluid
DPI: days post injury
GFAP: glial fibrillary acidic protein
IHC: immunohistochemical staining
SCI: spinal cord injury
